# Is COVID-19 impacting cancer screening in Pakistan? An observational study of cancer screening test requests during the pandemic

**DOI:** 10.1016/j.amsu.2021.102934

**Published:** 2021-10-13

**Authors:** Dr Sibtain Ahmed, Dr Muhammad Abbas Abid, Ms Noureen Niaz Ali, Mr Junaid Ahmed, Dr Imran Siddiqui

**Affiliations:** a Department of Pathology and Laboratory Medicine, Aga Khan University. Stadium Road, Karachi, 74800, Pakistan; b Section of Clinical Chemistry, Department of Pathology and Laboratory Medicine, Aga Khan University, Stadium Road, P.O. Box 3500, Karachi, 74800, Pakistan; c University of Kentucky, United States; d Professor, Department of Pathology and Laboratory Medicine, Aga Khan University, Stadium Road, P.O. Box 3500, Karachi, 74800, Pakistan

## Abstract

**Background:**

The purpose of this study is to assess how the COVID-19 pandemic affected cancer screening at a large tertiary care setting in the city of Karachi, the third largest city in the world, and to identify if there has been any decrease in cancer screening during the ongoing pandemic.

**Methods:**

A retrospective observational study was conducted at the clinical chemistry laboratory at the Department of Pathology & Laboratory Medicine, Aga Khan University Hospital (AKUH), Karachi Pakistan. Data for test volumes was extracted from the Integrated Laboratory Management System (ILMS) for the following tumor markers: CA19 Carbohydrate Antigen 19-9 (CA 19-9), Calcitonin, Prostate Specific Antigen (PSA), from 2017 to 2020. Data from January 1st, 2017 till December 31st, 2019 was recorded and compared with the test volume data from January 1st, 2020 till December 31st, 2020. Number of tests performed in the prior 3 years were compared with tests performed in 2020, specifically looking at changes during the lockdown period in 2020 (1st March – 9 th April) and compared with the same period in preceding years.

**Results:**

During the four-year period, a total of 6,530 tests were performed for CA19-9, 893 for Calcitonin, and 54,769 for PSA. Year 2019 recorded the highest volume for all 3 tests with test volumes increasing continuously from 2017 to 2019. Number of tests performed decreased throughout the year 2020 for Calcitonin and PSA, whereas volume of tests for CA19-9 only reduced during the lockdown period while increased in the non-lockdown period as compared to previous years. Highest percent decline during the 2020 lockdown period was seen for Calcitonin (-62.5%), followed by PSA (-51.8%) and CA19-9 (-19%).

**Conclusion:**

In conclusion, the amount of CA19-9, Calcitonin, and PSA tests performed in Karachi, Pakistan has drastically reduced due to the lockdown that was mandated due to the COVID-19 outbreak. It is crucial that despite an imposed lockdown, regular cancer screening must continue.

## Highlights

• Lockdowns and general fear of visiting hospitals during COVID-19 impacted cancer screening in Pakistan.
• A comparison of lab test requisitions from January 2017–December 2019 and January–December 2020 was undertaken.
• CA19-9, Calcitonin, and PSA screening tests reported a major decline.
• Highest percent decline during the 2020 lockdown period was seen for Calcitonin (−62.5%).

## 1 Introduction

COVID-19 pandemic has caused unprecedented morbidity and mortality globally [1]. Though the direct effects of COVID-19 are well studied and documented, the indirect losses incurred financially, and non-COVID related health issues are remain under-reported [2]. The lockdowns imposed by the governments to curb the spread of the disease resulted in significant financial losses in terms of businesses, jobs and travel restrictions. Although some of these losses were predicted beforehand and prepared for, healthcare sector, apart from COVID-19, is one area that may have been overlooked.

Literature suggests multi-faceted consequences of COVID-19, including exacerbation of pre-existing diseases [3]. One of the disease groups where the effects of pandemic could be particularly detrimental is cancer, specifically cancer screening. With over 19 million new cancer cases in 2020 and almost 10 million cancer deaths; this group cannot be overlooked irrespective of the pandemic situation [4]. COVID-19 pandemic could be harmful for such patients if it results in delayed diagnoses and/or treatment. Travel restrictions, lockdowns imposed by governments and general fear of visiting hospitals during acute rise in COVID-19 cases may interfere with diagnosis and treatment of cancer patients. Specially for cancer screening, delayed diagnosis can lead to advanced tumor stages at first diagnosis, which leads to poorer treatment outcomes and increased morbidity and mortality in such patients [5]. Pakistan being a low-middle-oncome country (LMIC), where medical resources are inadequate and healthcare facilities are scarce, maybe at an additional danger of delayed diagnosis of cancer patients.

The purpose of this study is to assess how the COVID-19 pandemic affected cancer screening at a large tertiary care setting in the city of Karachi, the third largest city in the world, and to identify if there has been any decrease in cancer screening during the ongoing pandemic.

## 2 Methods

A retrospective observational study was conducted at the clinical chemistry laboratory at the Department of Pathology & Laboratory Medicine, Aga Khan University Hospital (AKUH), Karachi Pakistan, which serves as a national reference lab for the country. With a large network of more than 290 phlebotomy centers and outreach laboratories spread across the country, it accommodates test requests from all the provinces. The laboratory operates to highest standards of quality and was the first lab to be accredited by College of American Pathologists (CAP) as well as Joint Commission International Accreditation (JCIA).

Data for test volumes was extracted from the Integrated Laboratory Management System (ILMS) for the following tumor markers: CA19 Carbohydrate Antigen 19–9 (CA 19–9), Calcitonin, Prostate Specific Antigen (PSA), from 2017 to 2020.

Data from January 1st, 2017 till December 31st, 2019 was recorded and compared with the test volume data from January 1st, 2020 till December 31st, 2020. No patient-related information was gathered hence ethical approval was not required.

Frequencies and means were calculated for the volume of tests performed during the 4-year period. Number of tests performed in the prior 3 years were compared with tests performed in 2020, specifically looking at changes during the lockdown period in 2020 (1st March – 9th April) and compared with the same period in preceding years. The changes in the number of tests performed were expressed in terms of percent change. Microsoft Excel 2019 was used for data analysis.

## 3 Results

During the four-year period, a total of 6530 tests were performed for CA19-9, 893 for Calcitonin, and 54,769 for PSA. Year 2019 recorded the highest volume for all 3 tests with test volumes increasing continuously from 2017 to 2019. Number of tests performed decreased throughout the year 2020 for Calcitonin and PSA, whereas volume of tests for CA19-9 only reduced during the lockdown period while increased in the non-lockdown period as compared to previous years. Highest percent decline during the 2020 lockdown period was seen for Calcitonin (−62.5%), followed by PSA (−51.8%) and CA19-9 (−19%). The means of tests along during the lock down period and the non-lockdown period, along with the percent change are given in Table 1.

**Table 1:** Comparison of tests performed in 2020 with mean tests performed 2017–2019.

	Tests performed in 2020	Mean tests performed (2017–2019)	Percent change
CA19-9			
Total period	1798	1577.33	12.3%
Lockdown period^a^	145	179	**−19%**
No lockdown period	1653	1398.33	15.4%
**CALCITONIN**			
Total period	211	227.33	**−7.2%**
Lockdown period^a^	12	32	**−62.5%**
No lockdown period	199	195.33	1.8%
**PSA**			
Total period	12778	13997	**−8.7%**
Lockdown period^a^	759	1576	**−51.8%**
No lockdown period	12019	12421	**−3.2%**

a Lockdown period was between March 1 and April 9, 2020.

## 4 Discussion

The results of our study show a decrease in cancer screening tests during the pandemic, especially during the lockdown period. This could result in delayed diagnosis and treatment of cancer patients which can be detrimental for patients already facing a global catastrophe.

The results are in-line with findings from other regions as well. Patt et al. performed a study in US population and reported that at the peak of pandemic in April 2020, screenings for breast, colon, prostate, and lung cancers were lower by 85%, 75%, 74%, and 56%, respectively [6]. They reported that institutional providers had greater reductions in delivery of cancer care. Similar results were reported by Ferrari et al. from Italy who reported a 62% decrease in PSA tests requests during the lockdown period [7].

Although the results in our study are in-line with the results from other regions; the consequences could be more severe for Pakistan due an already low cancer screening as compared to developed countries. The CA19-9 tests volumes, apart from the lockdown period, saw a rebound increase in 2020 as compared to previous years. This increase can be attributed to tests that were not performed during the lockdown period. Also, the tests performed each year has been continuously increasing from 2017 onwards. Surprisingly, no such rebound increase was seen for Calcitonin and PSA, which could be alarming, if the cancer screening was never performed on such patients.

The overall decrease in cancer screening tests performed could be attributed to a number of reasons. One reason that could be the fear of leaving the house during the lockdown period. A study conducted March 2020 in Karachi, Pakistan showed that 62.5% of respondents claimed to have feelings of anxiety due to the pandemic [8]. Another reason could be logistic difficulties due to closure of public transportation during lockdown. This made it harder for patients relying on public transportation to access healthcare facilities [9]. Another reason may be possible confusion on whether testing services were still available. The pandemic caused many businesses and clinics to suspend operations. This may have led to assumption that cancer screening services were not available during the lockdown period [10]. The poverty level of Pakistan is projected to rise about 33.7% due to the pandemic [11]. This could also be a reason for decreased testing during the pandemic period.

The limitation of our study is a single center design. Also, only 3 cancer screening tests were studied. But this was a first of its kind study from Pakistan which can give insight about impact of COVID-19 on healthcare delivery in the region. The results of the study can be used to educate patients and healthcare providers about the potential gaps created in healthcare delivery and address them using structured educational approaches so essential health work in not missed.

## 5 Conclusion

In conclusion, the amount of CA19-9, Calcitonin, and PSA tests performed in Karachi, Pakistan has drastically reduced due to the lockdown that was mandated due to the COVID-19 outbreak. It is crucial that despite an imposed lockdown, regular cancer screening must continue.

## Annals of medicine and surgery

The following information is required for submission. Please note that failure to respond to these questions/statements will mean your submission will be returned. If you have nothing to declare in any of these categories then this should be stated.

## Please state any conflicts of interest

None.

## Please state any sources of funding for your research

None.

## Ethical approval

Not applicable as the research work is based on trend analysis of lab test request of cancer screening laboratory tests and does not involve intervention/interaction with human subjects or related information.

## Consent

Not applicable.

## Author contributions

SA conceived the idea and drafted the manuscript. MAA performed the data analysis and write-up. NNA was involved in data collection and analysis. JA assisted in writing the first draft and performed the data cleaning. IS reviewed the manuscript for intellectual content and critically appraised the final draft. All the authors have accepted responsibility for the entire content of this submitted manuscript and approved submission.

## Registration of research studies

Not applicable.

## Guarantor

Dr Sibtain Ahmed.

Assistant Professor, Department of Pathology and Laboratory Medicine.
